# Root–Soil Interactions for Pepper Accessions Grown under Organic and Conventional Farming

**DOI:** 10.3390/plants12091873

**Published:** 2023-05-03

**Authors:** Ivan I. Morales-Manzo, Ana M. Ribes-Moya, Claudia Pallotti, Ana Jimenez-Belenguer, Clara Pérez Moro, María Dolores Raigón, Adrián Rodríguez-Burruezo, Ana Fita

**Affiliations:** 1 Instituto de Conservación y Mejora de la Agrodiversidad Valenciana, Edificio 8E Escalera J, CPI, Universitat Politècnica de València, 46022 Valencia, Spain; ivmoman@posgrado.upv.es (I.I.M.-M.);; 2 Centro Avanzado de Microbiología Aplicada, Universitat Politècnica de València, Camino de Vera s/n, 46022 Valencia, Spain

**Keywords:** rhizosphere, soil respiration, enzymatic activity, N cycle, *Capsicum annuum*, soil microorganism

## Abstract

Modern agriculture has boosted the production of food based on the use of pesticides and fertilizers and improved plant varieties. However, the impact of some such technologies is high and not sustainable in the long term. Although the importance of rhizospheres in final plant performance, nutrient cycling, and ecosystems is well recognized, there is still a lack of information on the interactions of their main players. In this paper, four accessions of pepper are studied at the rhizosphere and root level under two farming systems: organic and conventional. Variations in soil traits, such as induced respiration, enzymatic activities, microbial counts, and metabolism of nitrogen at the rhizosphere and bulk soil, as well as measures of root morphology and plant production, are presented. The results showed differences for the evaluated traits between organic and conventional management, both at the rhizosphere and bulk soil levels. Organic farming showed higher microbial counts, enzymatic activities, and nitrogen mobilization. Our results also showed how some genotypes, such as Serrano or Piquillo, modified the properties of the rhizospheres in a very genotype-dependent way. This specificity of the soil–plant interaction should be considered for future breeding programs for soil-tailored agriculture.

## 1. Introduction

Capsicum peppers are one of the most relevant vegetables (and spices) in the world. They are an economically important crop, particularly appreciated for their nutritional properties and antioxidant content [1]. In Spain, which is the main pepper producer within the EU [2], peppers are cultivated mainly as an intensive high input crop in the Andalucía and Murcia region [3]. However, in recent years, organic farming has increased in importance [4]. In contrast with conventional farming, where production is based on creating an “ideal” environment for plant development by limiting abiotic or biotic restrictions by any means, organic agriculture is based on maintaining an equilibrated ecosystem (especially in the soil, as no other substrates are allowed) compatible with agricultural production. Therefore, their management and consequences for the environment can be very different.

It is well known that plant characteristics, development, yield, and quality are affected by the environment [5,6,7]. The majority of research focuses on temperature or hydrological conditions, but little is known about the soil environment, which can be profoundly different from one field to another, especially if they are under distinct management systems. Different soil-environment conditions affect nutrition [8] and the health condition of the soil. For example, the effect of plant growth-promoting rhizobacteria (PGPR) in eliciting so-called “induced systemic tolerance (IST)” in plants under different abiotic stresses is well known [9,10,11]. Most of these interactions take place at the rhizosphere level.

The rhizosphere is the biologically active zone of soil where plant roots and soil interact and is of great importance for plant performance as well as for nutrient cycling and ecosystem functioning [12]. Rhizosphere processes are poorly understood and in situ agricultural soils are largely uncharacterized [13]. The rhizosphere dynamics involve complex interactions among roots, root exudates, the physical and chemical properties of the soil, and soil microorganisms, among others. All these factors change according to the others in the soil system. First, root architecture and root exudates depend mainly on plant species, genotype, and farming techniques, especially fertilization regimes [14,15]. It has been demonstrated how root exudates can shape the microbial community in the rhizosphere [16]. Second, different farming systems also modify the rhizosphere’s microbial communities [17]. For example, chemical fertilizers used in conventional farming supply nutrients, mainly N, P and K, whereas organic fertilizers also supply different amounts of C with macro and micronutrients, thus selecting microbial communities with different nutritional requirements [18,19]. Therefore, the different techniques applied (tilling, inorganic fertilization, etc.) modify the soil properties and microbiome [20]. Finally, soil microbes can play an important role in growth and nutrient uptake by plants as well as modifying their tolerance to biotic and abiotic stresses. Genotypes, farming systems, and microorganisms shape the performance of a crop in the field; thus, they are important aspects to consider in plant breeding.

Microbial community diversity and soil functional diversity are effective measures to illustrate the effects of natural and anthropological actions on the soil [21,22]. The characterization of soil microbiological community diversity and functional diversity can be assessed by different means, such as determining the microbial profiles [23], analyzing microbial catabolic potential [24], analyzing soil enzymatic activities [25], and by substrate-induced respiration measures [26]. Assessments of microbial activity with the MicroResp^TM^ system need small volumes of soil, making the method suitable for rhizosphere studies [27]. These traits can be used as appropriate indicators of soil heterogeneity and performance, which have been used extensively to check the health status of soil under different agricultural systems [28].

To sum up, to change from intensive agricultural systems to less harmful agriculture, increased knowledge of the rhizosphere’s processes is needed. In this paper, four accessions of pepper, selected for having diverse root systems and phosphorous uptake efficiency [14], are studied at the rhizosphere and root level under two farming systems (organic and conventional) to better understand root–soil interactions for more sustainable agriculture.

## 2. Results

**Figure 1:**
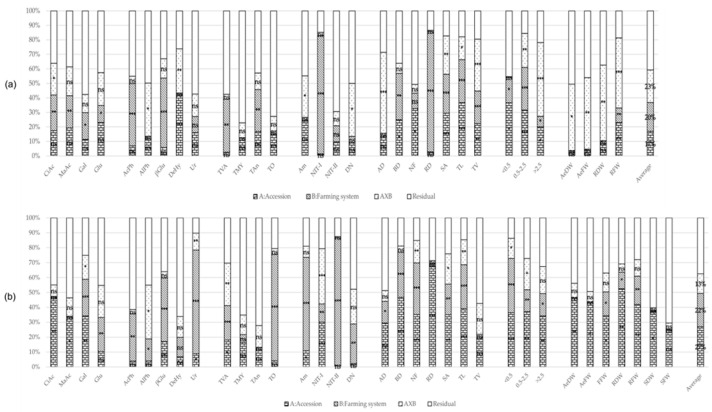
Percentage of MANOVA effects’ sum of squares (SS) contribution for trait; (**a**) At the vegetative stage (T1); (**b**) At the late fruit stage (T2). Percentage contribution of variance of each effect: Accessions and bulk (when available) (A, bricks pattern), Farming system (B, lines pattern), A × B interaction (dots pattern) and Residual (plain white). Substrate induced respiration: CiAc, MaAc, Gal and Glu; Enzymatic activity: AcPh, AlPh, BGlu, DeHy, and Ur; Microbial count: TVA, TMY, TAn, and TO; N_-_ cycle: Am, NIT-I, NIT-II, and DN; Root traits: AD, BD, NF, RD, SA, TL, TV, and diameter classes: ≤0.5, 0.5–2.5, and >2.5; Biomass: AeDW, AeFW, FFW, RDW, RFW, SDW, and SFW; Average percentage MANOVA effects’ SS; *–***: significant at *p*-values of 0.05–0.001, ns non-significant.

All data obtained were subjected to exploratory MANOVA to identify the possible effects of the accessions and the farming system on the rhizosphere’s characteristics and plant performance. This analysis was carried out by separating the two sampling times, which correspond with two phenological stages of the plant: at the vegetative stage (T1, summer) and at the late fruit stage (T2, autumn). The MANOVA demonstrated that the farming system had a significant effect on the rhizosphere’s studied traits at T1 (Figure 1a). At this time, 9 out of 17 evaluated rhizosphere traits showed significant differences due to the farming system, in contrast with just 1 trait that showed significant differences according to the accession. Interestingly, accession × farming system interaction was observed in five studied traits. This situation changed at T2 (Figure 1b), where although the farming system effect was the highest, being significant for 12 out of 17 traits, the accession effect increased the number of meaningful traits, being significant for 7 out of 17 traits. In this phenological stage, the interaction effect was significant for five traits again, but only one was the same as T1. In the case of root and biomass traits, influence at T1 of the accession was as important as the farming system and interaction effects, being significant in 9, 9, and 10 out of 14 traits, respectively (Figure 1a). At T2, the accession and farming system effects increased (15 and 11, respectively, Figure 1b). In the following sections, the values of the different rhizosphere and plant traits in each farming system and sampling time are described.

### 2.1. Rhizosphere’s Traits

#### 2.1.1. Substrate Induced Respiration

At T1, induced respiration was significantly different between farming systems regardless of the substrate (Figure 1a and Table 1). Induction with citric acid (CiAc) and malic acid (MaAc) produced lower respiration under the organic farming system compared to the conventional farming system: 17.70 µg CO_2_ g soil^−1^ h^−1^ at organic vs. 53.02 µg CO_2_ g soil^−1^ h^−1^ at conventional, 21.47 µg CO_2_ g soil^−1^ h^−1^ at organic vs. 47.02 µg CO_2_ g soil^−1^ h^−1^ at conventional, respectively (Table 1). CiAc also showed accession × farming system interaction, probably due to the different performance of *Bola* accession, which has higher respiration in organic, 45.82 µg CO_2_ g soil^−1^ h^−1^, than conventional soil, 38.19 µg CO_2_ g soil^−1^ h^−1^.

Respiration with sugar substrates such as Gal and Glu was also lower in the organic field than the conventional (1.58 µg CO_2_ g soil^−1^ h^−1^ vs. 2.96 µg CO_2_ g soil^−1^ h^−1^ and 4.02 µg CO_2_ g soil^−1^ h^−1^ vs. 13.80 µg CO_2_ g soil^−1^ h^−1^, respectively). None of the accessions showed significantly different respiration rates from the ones shown in the bulk soil.

At T2, the trend for all the substrates in the organic system was to show a slight increase with respect to T1 (Table 1), whereas in the conventional system, the induced respiration was lower at T2 than at T1, especially for Gal and Glu. Therefore, the induced respiration for CiAc and MaAc at T2 was not significantly different between farming systems, but there were significant differences for Gal: 1.83 µg CO_2_ g soil^−1^ h^−1^ at organic vs. 1.15 µg CO_2_ g soil^−1^ h^−1^ at conventional, and for Glu: 4.73 µg CO_2_ g soil^−1^ h^−1^ at organic vs. 2.61 µg CO_2_ g soil^−1^ h^−1^ at conventional (Figure 1b, Table 1). Interestingly, at T2, the accession factor was the major contributor to the variance for CiAc, MaAc, and Gal (Figure 1b). For these sources of carbon, the rhizospheres of *Serrano* and *Bola* produced significantly higher amounts of CO_2_ than the rest of the accessions, both in conventional and organic farming systems (Table 1) and were stable between phenological stages.

#### 2.1.2. Soil Enzymatic Activity

Acid phosphatase activity (AcPh) was significantly different among farming systems at both phenological stages (Figure 1a,b), with mean values significantly higher at organic (91.09 µmol g^−1^ h^−1^ at T1 and 89.88 µmol p-nitrophenol soil g^−1^ h^−1^ at T2) than at conventional (73.89 µmol g^−1^ h^−1^ and 74.51 µmol g^−1^ h^−1^ at T1 and T2, respectively) (Table 2). No significant differences were observed between the rhizospheres or the bulk soil for this enzymatic activity. The case of alkaline phosphatase (AlPh) was different. At T1, the only significant effect was the accession × farming system interaction (Figure 1a), which was due mainly to the higher AlPh activity registered for *Serrano*’s rhizosphere in comparison to the rest of the accessions or the bulk soil at organic (563.92 µmol p-nitrophenol soil g^−1^ h^−1^) but not at conventional (402.87 µmol g^−1^ h^−1^). This trend was conserved at T2, with significant differences in AlPh activity in the rhizosphere of *Serrano* (569.41 µmol g^−1^ h^−1^ at organic). In addition, at T2, there were significant differences between farming systems for AlPh (485.33 µmol g^−1^ h^−1^ at organic and 427 µmol g^−1^ h^−1^ at conventional, Table 2).

β-glucosidase (βGlu) activity was similar at both sampling times; the activity of this enzyme was significantly higher in organic 0.72 µmol p-nitrophenol soil g^−1^ h^−1^ at T1 and 0.62 µmol g^−1^ h^−1^ at T2 than for conventional cultivation, 0.42 µmol g^−1^ h^−1^ at T1 and 0.45 µmol g^−1^ h^−1^ at T2. For this enzyme activity, there were no differences regarding the accession rhizospheres. Dehydrogenase (DeHy) activity in the soil was around 1 µmol INTF soil g^−1^ h^−1^. At T1, there were accession and accession x farming system interaction effects, mainly due to DeHy activity in the *BOL-58* rhizosphere, 1.18 µmol g^−1^ h^−1^, in the organic field, which was higher than other genotypes and bulk soil, and by the low activity of DeHy in the rhizosphere of *Serrano* in the conventional field, 0.78 µmol g^−1^ h^−1^ (Table 2). At T2, there were no significant effects. The activity of the urease (Ur) was around 0.165 µmol N-NH_4_^+^ soil g^−1^ h^−1^ T1 and there were no differences due to the farming system or accession. However, at T2, although the activity was similar, an increase was observed for all accessions and bulk, as well as significant differences among the two farming systems (0.36 µmol g^−1^ h^−1^ at organic and 0.20 µmol g^−1^ h^−1^ at conventional). For the accession, Ur activity from *Bola*’s rhizosphere was higher than the rest in organic cultivation, at 0.47 µmol g^−1^ h^−1^ (Table 2).

#### 2.1.3. Microbial Counts

At T1, the counts of total viable aerobic mesophilic bacteria (TVA) and the counts of total anaerobic bacteria (TAn) were significantly different depending on the farming system (Figure 1a). The higher means were observed for the organic system, with 7.40 log CFU g soil^−1^ for TVA and 6.45 log CFU g soil^−1^ for TAn (Table 3). For total molds and yeast counts (TMY) and for total oligotrophic bacteria counts (TO), no significant effects or differences were observed among systems, nor between the rhizosphere’s accessions or the bulk soil, with the average count for molds and yeast being 5.56 log CFU g soil^−1^ and the average count for oligotrophic bacteria being 7.27 log CFU g soil^−1^. At T2, the difference among systems for the TVA was maintained as in T1 (Figure 1b, Table 3). However, during this period, the accession and accession x system interactions also had significant effects. The rhizosphere of *Serrano* in the conventional system (7.79 log CFU g soil^−1^) had higher counts of viable bacteria than *Piquillo*’s rhizosphere (6.76 log CFU g soil^−1^) (Table 3). No differences were observed due to the farming systems, rhizospheres, or bulk soil at T2 for TMY (5.21 log CFU g soil^−1^) and for TAn (5.92 log CFU g soil^−1^). It is interesting to note that the TAn was lower at T2 in organic farming but not in conventional farming. Finally, TO showed significant differences among systems (Figure 1b, Table 3) at T2. In this case, the conventional system showed lower counts (6.66 log CFU g soil^−1^) in comparison with the organic system, or the values registered at T1.

#### 2.1.4. Nitrogen Catabolism Potential (N-Cycle)

At T1, for ammonification (Am), the only significant effect was the accession × farming system interaction (Figure 1a), mainly due to the rhizosphere of *Piquillo* in the conventional field (310.4 mg NH_4_^+^-N g^−1^), which showed a higher rate of ammonification in comparison with the others, contravening the general tendency to have higher ammonification potential in the organic field (Table 4). At T1, the nitrification I (NIT-I) potential was significantly higher in the organic (20.27 mg NO_2_-N g^−1^) than the conventional (2.56 mg NO_2_-N g^−1^). No differences were found among samples for nitrification II (NIT-II), with an average of 20.34 mg NO_3_-N g^−1^. For potential denitrification (DN), there were no significant differences among samples, which were, on average, 2.8.

At T2, all N-cycle parameters showed differences among farming systems with higher activity in the organic field, except for DN, which had higher levels in the conventional field (Figure 1b, Table 3). Interestingly, again, the rhizosphere of *Piquillo* in conventional farming showed the highest Am among accessions and bulk (31.04 mg NH_4^+^_-N g^−1^). For NIT-I, the general values were very low, ranging from 0.15 to 0.76 mg NO_2^−^_-N g^−1^, exceeding the values of the rhizosphere of *Bola* with values of 2.53 mg NO_2^−^_-N g^−1^, although this value is lower than in T1. Lastly, at T2, there were significant differences in DN levels, which were higher in the conventional field (Table 4).

### 2.2. Plant’s Traits

#### 2.2.1. Biomass and Yield

At T1, there were significant accession × system interaction effects for all biomass traits (Figure 1a). For organic fields, *BOL-58* and *Serrano* always had heavier shoots (AeDW for dry and AeFW for fresh matter) and roots (RDW for dry and RFW for fresh matter); for the conventional field, *Bola* and *Piquillo* were the heaviest and significantly different (Table 5).

At T2, there were significant differences among accessions for almost all biomass traits (Figure 1b, Table 6). *BOL-58* stood out for its great vegetative growth in the aerial part (AeFW of 1103 g at organic and 1533 g at conventional), whereas *Serrano* had the heavier root system (RFW of 33.69 g at organic and 23.9 g at conventional). There were no significant differences in the development of the aerial parts of the accessions depending on the farming system (Figure 1b, Table 5); however, the roots of all genotypes were heavier in the organic field than in the conventional field (Table 5). Contrarily, the total yield (FFW for fresh matter) was higher in conventional than in organic for the accessions tested, except for *Serrano*. There were no significant differences in the stump weight (SDW for dry and SFW for fresh matter) in any accession or farming system (Figure 1b, Table 5), but *Serrano* had the highest values, as seen with root weight.

#### 2.2.2. Root Parameters

At T1, there were significant differences in the roots among accessions, but in most cases, there were also differences due to the farming system and, in some cases, interactions (Figure 1a). The general trend according to the farming system was for the roots to be more branched (0.95 forks cm^−1^) and dense (0.33 g cm^−3^) in the organic field than in the conventional field (Table 6), but to be shorter with less volume. However, the roots of the plants growing in the organic system were longer, with a diameter higher than 2.5 mm (>2.5 class).

Regarding the performance of the genotypes at T1, *Bola* and *Piquillo* showed significantly longer (TL, 2356 cm, and 1650 cm, respectively) and more voluminous roots with a higher number of forks than the other genotypes, especially in conventional conditions (NF, Table 6) in the conventional field. *Serrano* stood out for its great root diameter (AD: 1.06 mm) and longer thicker roots (>2.5: 25.11 cm) in the organic field.

At T2, the situation was different, and in this case, higher values were found not only for branching parameters (BD, NF) but also length (TL, >0.5, 0.5–2.5, and >2.5), root volume (TV, cm^3^), and root area (SA, cm^2^) in organic farming (Table 6). There were also differences among accessions and accession x farming system interactions. At this time, *Piquillo* performed the best in terms of root length (TL) and branching (NF); however, this higher performance was more accentuated in organic farming. Again, like T2, *Serrano* had a vast root diameter (AD) and thicker roots (>2.5).

### 2.3. Correlations and Exploratory Factor Analysis

#### 2.3.1. Correlation among Root, Biomass, and Rhizosphere Traits

**Figure 2:**
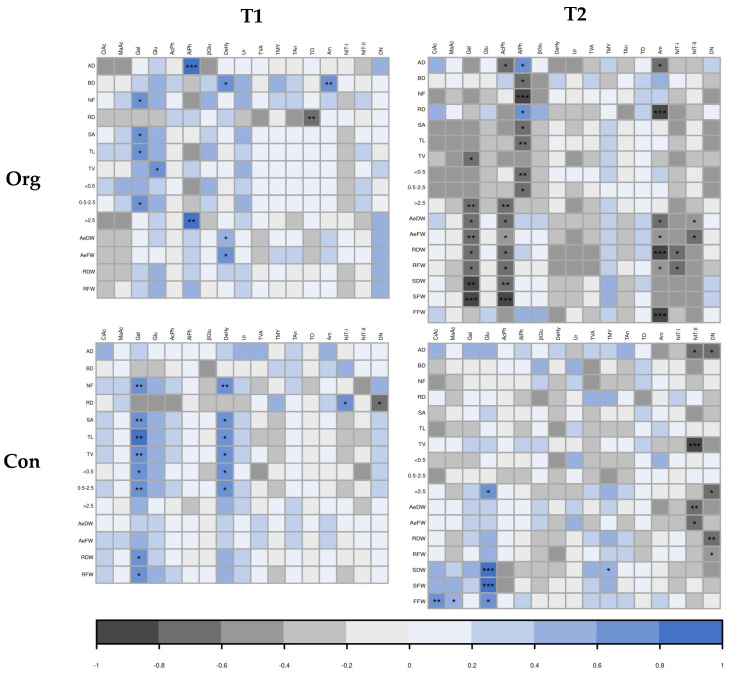
Heatmap showing correlations among plant traits and rhizosphere traits: citric acid (CiAc), malic acid (MaAc), galactose (Gal), glucose (Glu), acid phosphatase (AcPh), alkaline phosphatase (AlPh), β-glucosidase (βGlu), dehydrogenase (DeHy), and urease (Ur), total viable aerobic mesophilic bacterial count (TVA), total molds and yeast count (TMY), total anaerobic bacteria (TAn), total oligotrophic bacterial count (TO), ammonification potential (Am), nitrification I potential (NIT-I), nitrification II potential (NIT-II), denitrification potential (DN) in two farming systems (organic; org and conventional; con) at two sample times (T1: summer, T2: autumn). Pearson’s multiple correlation coefficient (−1 to 1 range, dark-gray to blue scale), in which the significance level of the *t*-test at 5%, 1%, and 0.1% was evaluated. The significance levels were indicated by *, **, and ***, respectively.

To better track root–soil interactions, a correlation analysis was carried out (Figure 2). Some interesting correlations between plant traits and rhizosphere parameters were observed (Figure 2). Interestingly, these correlations varied depending on the sampling time and farming system. In the first place, the respiration of the rhizospheres using Gal as the substrate was positively correlated at T1 and in both farming systems with many root parameters, mainly related to the root length and weight. This situation changed at T2, where the correlations were different for each farming system; in the organic field, respiration induced with galactose was negatively correlated with higher plant biomass, whereas in the conventional field, plant parameters were uncorrelated with plant traits.

At T1, AlPh was positively correlated only in the organic field, with a diameter of the root (AD and >2.5). DeHy activity was positively correlated with different plant traits, depending on the system. In the organic field, it was positively correlated to BD and biomass traits, whereas in the conventional field, it was correlated with NF and length parameters (TL, TV, <0.5 and 0.5–2.5). At T2, the only enzymatic activities that significantly correlated with root traits were AcPh and AlPh, and only in organic soil. AcPh activity was correlated negatively with lower biomass, whereas AlPh activity was correlated negatively with lower diameter classes of root length but positively with AD and RD.

In general, the microbial count was not correlated with any plant parameter at any time or farming system, but a correlation was observed with the higher oligotrophic microorganisms (TO) with less dense roots (RD) (Figure 2).

The nitrogen cycle parameters showed very few correlations with plant traits at T1 (Figure 2). Nevertheless, a negative correlation between Am potential and BD for organic farming, a positive correlation between RD and NIT-I potential, and a negative correlation between RD and DN potential were observed. At T2, more significant correlations were observed. In this case, AM potential was correlated with thinner (AD) and less dense roots (RD). This happened only in the case of the organic field, where Am, NIT-I, and NIT-II were also somehow correlated with lower values of biomass and production (AeDW, AeFW, RDW, RFW, and FFW). This negative correlation was only maintained in the conventional farming system for shoot biomass (AeDW and AeFW) and the NIT-II potential of the soil. In this farming system, interestingly, the root diameter (AD), the length of the thick roots (>2.5), and the root biomass (RDW and RFW) were negatively correlated with DN potential.

#### 2.3.2. Factor Analysis of Rhizosphere and Bulk Soil

To resume all possible correlations among soil traits, an exploratory factor analysis was carried out, where factor load was considered significant at levels higher than 0.4. For the analysis, bulk samples were taken for both farming systems and sampling times; all root and biomass traits were excluded from the analysis. At T1, fifteen traits were factorizable (through the overall Measure of Sampling Adequacy, or MSA, at least 0.6 [29], Table 7) in three factors. At T2, sixteen traits were factorizable, one more (DeHy) than at T1.

**Figure 3:**
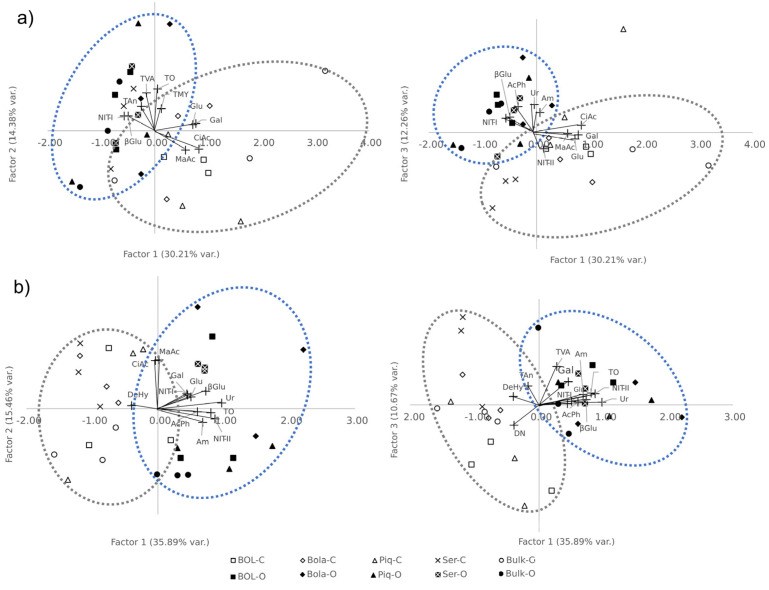
Varietal projection on the new factorial space. Scorings of accessions, bulks, and trait loads. (**a**) in vegetative stage (T1), left is first and second factors projection; right is first and third factors projection. (**b**) in late fruiting stage (T2), left is first and second factors projection; right is first and third factors projection. Blue dotted line group are mostly organic farming system samples, and gray dotted line group are mostly conventional farming system samples.

At T1, the main loads for the first factor (which explains the 30.21% variation, F1.1) were all the induced respiration substrates (CiAc, MaAc, Gal, and Glu) with positive loads, and βGlu activity and NIT-I with negative loads (Table 7). The main loads for the second factor (14.38% of the variation, F2.1) were all the microbial counts with positive loads (Table 7). For the third factor (12.26% of variance explained, F3.1), the main loads were obtained with AcPh, βGlu, Ur, and Am potentials with positive loads and NIT-II with a negative load. When observing the projection (Figure 3) of the rhizosphere’s scores of each accession and bulk sample in each factor (Table S1, Supplementary Data), first (F1.1) and second (F2.1) factors grouped each FS, being conventionally characterized by higher respiration rates and lower βGlu activity and NIT-I potential (Figure 3). For *Serrano* rhizosphere samples with scores that were not differentiated between farming systems, these F1.1 and F2.1 traits had a strong genotype influence, therefore being grouped very closely regardless of the farming system.

At T2, the first factor (35.89% of the variation, F1.2; Table 7) was characterized by positive loads Gal, Glu, AcPh, βGlu, Ur, TO, Am, NIT-I, and NIT-II, and DeHy with a negative load (Table 7). The second factor (F2.2) had only two but very high loads, CiAc and MaAc. Finally, the third factor (F3.2) had three traits with positive loads (Gal, TVA, and Tan) and one with negative and significant loads (DN). At this time, again, we were unable to differentiate the F1.2 scores among farming systems (Figure 3). Additionally, with F2.2, carboxylic acid’s induced respiration weight interestingly differentiated all bulk and *Serrano* samples. F3.2 was able to differentiate a few accessions from the bulk samples; in the organic farming system, *BOL-58* and *Serrano* had positive scores, and in the conventional FS, *BOL-58* and bulk had negative scores (Table S1, Supplementary Data) (Figure 3).

## 3. Discussion

### 3.1. The Farming System Conditions the Status of the Soil

The results showed the complexity and dynamics of the soils tested. In this experiment, we selected two similar fields that were specifically chosen to minimize the effects of the climatological conditions and physicochemical properties of the soil. Both soils differ mainly in the management of the crop, with the application of chemical fertilizers and pesticides in the case of conventional farming and only organic matter amendments and organic agriculture-authorized products in the case of organic cultivation. The results showed clear differences in the studied soil traits, which we were able to differentiate among both soils from the very beginning of the experiment. This clearly showed the imprint of the historic records of the soil, which were somehow maintained through the season [30]. Olayemi et al. [31] in a 6-year study on loamy-silty soils of a semiarid climate from Colorado (USA) concluded that soil biological communities are generally enriched and more diverse under continuous organic residue retention, resulting in higher soil biodiversity and a range of critical soil functions mediated by soil organisms. The results obtained here for the different soil traits studied agree with that idea.

First, the induced respiration rate measured through the colorimetric method may be an indicator of the level of microorganism biomass and diversity in certain soils [27,32,33,34]. In addition, the characterization of soil microbial catabolic diversity through substrate-induced respiration could be a way to monitor soil biological resistance and resilience [35]. However, microbial respiration and carbon utilization are not static but variable throughout the year, probably due to seasonal variations in the characteristics of the studied ecosystems [36]. Therefore, the differences among the induced respiration results in the two sampling times in this experiment are not surprising at all, as sampling was carried out both at the beginning (T1: summer) and at the end (T2: autumn) of the warmest period of the year in Valencia (average T°, min and max = 24.3°, 9.3–37 °C). Previous studies carried out in drylands showed that soil microbial respiration can adapt to the environmental temperature through the physiological adjustment of individual or entire microbial populations [37]. In addition, this response of microbial communities to different temperatures could be used to predict climate-induced changes in carbon fluxes [38]. It has been found that increased temperature reduces total microbial biomass, but at the same time, the response to temperature is dependent upon substrate quality [39]. Differences in the temperature sensitivities of taxa and the taxonomic composition of communities determine community-assembled bacterial growth [40].

In the present experiment, the induced respiration rate in the organic soil was lower at the beginning of the experiment, whereas at T2, the respiration rates from the soils of the two systems were alike. The fact that the respiration rates were more stable in the organic field than in the conventional may indicate that the microbial populations are buffered throughout the warm season, probably due to their specific microbial profile, which may differ from the microbial community in the conventional plot. Despite the general trend observed through time according to the farming system, it is important to note that not all substrates behaved the same. Creamer et al. [41] demonstrated, after testing eight substrates in 81 soils, that the substrate behavior was dependent upon combinations of land-use, pH, and soil organic matter. Specifically, they reported greater utilization of carboxylic acid-based substrates in arable sites, which concords with our results, with higher respiration rates with CiAc and MaAc, especially in the conventional field. In addition, the soils assayed had a pH of 8.2, which has been reported as negative for the use of Gal but positive for the use of organic acids [41].

Secondly, other important indicators of soil quality are soil enzymes [42]. The different soil enzymatic activities are the result of proliferating microorganisms and the accumulation of enzyme action. The main sources of accumulated enzymes are the cells of microorganisms, and a small part may come from organic plant and animal residues. Dehydrogenase and β-glucosidase are generally used as indicators for microbial activity. Dehydrogenase is involved in intracellular oxidation-reduction processes, and β-glucosidase, as an extracellular enzyme, is fundamental in the hydrolysis and degradation of soil carbohydrates, releasing glucose. This represents the important contribution of energy to soil microorganisms. Alkaline and acid phosphatase are two non-specific enzymes that catalyze the hydrolysis of glycerophosphates and differ by their optimal pH for action, 11 and 6, respectively, and are involved in the release of P from organic forms. Finally, urease activity in soil may be associated with living cells, dead cells, or cell debris, or may even be immobilized in humic clays and colloids. Urease hydrolyzes urea into ammonium, a usable form of N by plants, and carbon dioxide, participating actively in the nitrogen cycle and then in the fertility of the soil [43]. The enzymatic activities observed in the soils analyzed here remained stable throughout the studied period, and were similar to those reported by other authors such as Jat et al. [44], who studied soils from the rhizosphere and bulk soils of cereal crops with different management in India. On the other hand, other authors reported that in Western Spain, for acid phosphatases, levels were more likely to be 10–40 μmol g^−1^ h^−1^ [45].

In addition to soil properties, soil management has been described as a driver of the soil enzymatic activity [46]. The results in this paper showed significant differences among the two-farming systems for all tested enzymes, except for dehydrogenase. Interestingly, of all the enzymes evaluated in this experiment, DHA was the only one found to have exclusive intracellular activity. This may indicate similar microbial mass among conventional and organic fields but different profiles of microorganisms. Moreover, as the fundamental enzyme for the carbon cycle in soil, on average, β-glucosidase activity was proximally 30% higher in organic soil, evidencing the importance of the presence of organic carbon in the soil, which was higher due to the manure fertilization in the organic field [47]. In fact, its higher content in organic matter has been continuously correlated with higher enzymatic activity. AlPh, AcPh, and urease were also significantly higher in the organic than conventional farming systems; these enzymes are influenced not only by the organic matter of the soil but also by the fertilization status. Higher fertilization has been correlated with lower activity of those enzymes, although in the case of acid phosphatase, it has been described that increasing levels of N produce higher activity [48].

Thirdly, microbial counts offer an opportunity to quantify the microbial biomass and its profile. In concordance with previous enzymatic activity, microbial counts were more abundant in the organic than conventional farming systems, with dependence on the community profile and the sampling time. For instance, total viable aerobic (TVA) counts were higher in organic than conventional at both sampling times, whereas anaerobic microbial counts were higher in organic only at T1 and for oligotrophs only at T2. Higher levels of microorganism diversity are usually reported in organic farming due to the higher amount of organic matter [49]. Additionally, shifts over time in microbial communities are common [50]. On the contrary, counts of molds and yeast were similar regardless of the farming system or sampling time. Other authors have pointed out that these communities are relatively stable, with a typical profile depending on the soil type and climate and a few genera dominating over the others [51,52].

Finally, as in the case of microbial counts, the results presented here also indicate differences in the N cycling dynamics among farming systems. Microorganisms have been controlling the Earth’s nitrogen cycle since life originated [53]. The nitrogen cycle refers to the dynamic process of circulating this element cyclically through the soil and the atmosphere, allowing the transformation of nitrogen into forms accessible to the metabolism of microorganisms, plants, and animals. Nitrogen fixation is the process of reducing molecular nitrogen to ammonia. The group of atmospheric nitrogen fixers consists of numerous organisms, including (i) aerobic nitro-gen-fixers from the genera *Azotobacter*, *Beijerinkia*, *Derexia* and *Azotomones*; (ii) strict anaerobic bacteria, such as those of the genus *Clostridium*; and (iii) symbiotic fixing bacteria such as *Rhizobium* for the Fabaceae family or *Frankia* for non-legume angiosperms. Mineralization is the process of transforming organic nitrogen into ammonia. The functional group of mineralizers is broad and includes fungi and bacteria. In our experiment, total molds and yeast and total anaerobic bacteria correlate with the potential mineralization rate. Some fungal genera such *Mucor* and *Rhizopus* or *Aspergillus* and *Penicillium* have been described as N mineralizers, whereas mineralizing bacteria could be represented by genera such as *Pseudomonas*, *Clostridium*, *Serratia*, *Bacillus*, *Escherichia* and *Micrococcus* [54]. In the nitrification process, microorganisms convert ammonium to nitrate to obtain energy. In our experiment, total oligo-tropic counts and anaerobic correlates with the nitrification process. Typical nitrificating bacteria belong to the family *Nitrobacteriaceae: Nitrosomonas*, *Nitrosobolus*, *Nitrosospira*, *Nitrosococcus*, and *Nitrosovibrio*, oxidizing ammonium nitrogen to nitrite and *Nitrobacter*, *Nitrospira* and *Nitrococcus* that oxidize nitrite to nitrate. Both mineralization and nitrification were higher in the organic system than the conventional one, indicating a higher ability to recirculate N among the system. Higher mineralization and nitrification rates were observed in organic farming systems described by some authors [55,56,57] due to the use by microbial communities of SOM (soil organic matter). The nitrification process is known to be enhanced when soil is warm (20–30 °C), which explains the significant drop in the nitrification potential of the soils at T2 (milder temperatures), which was more intense in the conventional than organic farming system.

Denitrification occurs when N is lost through the conversion of nitrate to gaseous forms of N, such as nitric oxide, nitrous oxide, and dinitrogen gas. In this experiment, conventional soil suffered higher denitrification, but the type of gas produced was not identified. Nitrous oxide is a potent greenhouse gas with a global warming potential [58], whereas N_2_ is inert. Lazcano et al. [59] pointed out the need to build up soil C stocks to contribute to N retention as microbial or stabilized organic N in the soil while increasing the abundance of denitrifying microorganisms and, thus, reducing the emissions of N_2_O by favoring the completion of denitrification to produce dinitrogen gas.

### 3.2. Rhizosphere Performance Depends on the Genotypes as the Crop Evolves

Despite the great importance of the soil properties and the farming system on the studied soil parameters, in this experiment, it was also possible to observe the influence of the plants on the rhizosphere properties, especially at T2. This showed that during a plant’s growth, it interacts with the surrounding environment in a very specific way. Rhizodeposits, root exudates, and root border cells shape microbial communities, pH, and other factors in the rhizosphere, thereby allowing plants to uptake a wider variety of nutrients for growth and inhibiting possible pathogens [15,60].

In this experiment, there were not many differences in the respiration rates between the rhizospheres of the accessions and the bulk soil, except for *Bola* and *Serrano*. Although other authors have also identified differences in rhizospheric catabolic activities at the species-dependent level [61], to our knowledge, this is the first time that such differences in respiration rates have been observed at the accession level.

Regarding enzymatic activity, generally, it is usually higher in the rhizosphere zone than in bulk soil due to the higher organic carbon deposition in this area, which creates favorable conditions for microbial activities [62]. Profuse vegetation, high root colonization, and no tillage have been correlated with greater soil enzyme activity [43,63,64]. Generally, rhizosphere soil is characterized by a higher amount of very labile carbon and lower contents of mineral nitrogen as well as other nutrients, with a 19–32 times higher number of microorganisms compared to bulk soil [65]. Contrary to what was expected, there were not a great deal of differences in this experiment among the samples on the rhizosphere or the bulk soil, except for the *Serrano* accession. In previous experiments, the action of the accessions on the enzymatic activity of the soils was more intense [66,67].

Exudates and other secondary metabolites have been described to alter the rhizosphere microbiota, as stated in a study by Hu et al. [68]. In our case, it was possible to observe this effect only for *Serrano* and *Piquillo*, which modified total viable counts at T2. The effect of certain plant growth-promoting rhizobacteria (PGPR) and the exudates of some accessions may be responsible for the increase in the total number of microorganisms observed, as other authors have described [12,13]. These results have shown that the rhizosphere communities modulated by the different exuded molecules make certain groups of microorganisms have more affinity for some genotypes than others [69]. Furthermore, in the case of *Piquillo* accession, N mineralization presented higher values in interactions in the conventional farming system. This ability to mobilize the N cycle seemed to be correlated with increasing aerial plant mass and fruit weight. There is clear evidence that plants are not passive conduits, taking up whatever N diffuses to their roots; instead, they can improve their N nutrition by (a) establishing symbiosis with soil microorganisms; (b) stimulating the activity of microorganisms in the root vicinity to increase N availability; and (c) increasing N conservation in soil by limiting microbial processes that lead to N losses, such as nitrification and denitrification, directly through the release of inhibitors from their roots [70,71].

### 3.3. Plant-Soil Interactions Are Complex and Multifactorial

*Piquillo*, *Serrano*, and *Bola* were selected to be part of this experiment due to their good performance for phosphorous acquisition in previous studies, either P uptake efficiency or P utilization efficiency. In those experiments, root length increased under P deficiency and fine roots were found to be correlated with P efficiency parameters [14]. Here, the capacity of these genotypes to alter the rhizosphere’s microbial community and function has been described for the first time, although the impact of such alterations on the actual nutrition status of the plant remains to be studied.

Contrary to what was expected, root morphological traits showed few correlations with the rhizosphere, being significant only for galactose and glucose-induced respiration, AcPh and AlPh, and some steps of the nitrogen cycle. Furthermore, the correlations were not regularly seen through the different farming systems and sampling times. Therefore, we can suggest that root exudation was more important than root morphology in creating differences among the accession’s rhizospheres. Unfortunately, the study of root exudates is difficult and still needs improvement [72].

Root exudation, root morphology, and mycorrhizal symbioses have been described as shaping belowground resource acquisition strategies in a species-dependent manner [73]. It seems that each plant species has its own strategy that favors one of the possible solutions over the others; for instance, the response of maize to P deficiency seems to be more dependent on root morphological changes than increasing root exudates [74]. The independence of the root morphology and the level of root exudates has also been described in studies such as that of Iannucci et al. [75], where they studied eight durum wheat genotypes for their root morphology, exudates, and soil community and only found a correlation between the last two. For future analyses, it would be convenient to study whether the root exudates are correlated with a certain root morphology, as some of our correlations may suggest, or whether they are totally independent.

## 4. Materials and Methods

### 4.1. Plant Material, Experimental Design, and Sampling

Four different pepper accessions, two Mediterranean (*Bola* and *Piquillo*) and two Latin-American (*BOL-58* and *Serrano*), were grown in an open field in the 2018 spring-summer season in two farming systems (FS): an organically managed field (FS-O) and a conventional field (FS-C), both located in Sagunto, Valencia, Spain, which belongs to a Mediterranean climatic area with hot and dry summers (Supplementary Table S2). Both fields were clay-loam with a pH of 8.22, EC 0.28 dS/m organic and 0.3 dS/m conventional, with a percentage of organic matter of 1.85% for organic and 1.65% for conventional. Both fields were furrow-irrigated with water from the same well. The fields were managed as in Ribes-Moya [5]. In the organic farm system, sheep manure (4 kg/m^2^) was applied as fertilizer at the beginning of the season. In the conventional field, there was one application of vegetable humus (4 kg/m^2^) and one application of a mix of nitrogen, phosphorus, and potassium (15-15-15) (50 g/m^2^) before transplanting, plus three foliar applications of calcium nitrate (10 g/L), and one application of iron chelate (3 kg/1000 m^2^) after transplanting. Pests and diseases were not treated in organic cultivation, whereas chlorpyrifos (48%, EC) and abamectin (1.8%, EC) were applied, combined with copper oxychloride (58.8% WP) as fungicide, in the conventional field. Adventitious plants were controlled mechanically at both sites. Three blocks of five plants per accession were randomly distributed in each field. Two phenological stages (PS) were also considered: at the end of the vegetative stage (T1) and the end of the fruiting stage (T2).

Rhizosphere soil samples (100 g) corresponding to each genotype were obtained as follows: at T1 and T2, the first 5 cm of topsoil of one plant per block was removed to avoid contamination from the surface. Then, the shovel was cleaned, the full plant was dug up to at least 20 cm depth, and the sample was taken carefully between the roots. Three bulk soil samples were taken with the same depth and procedure, but in the middle of the lanes, for each FS. All soil samples (rhizospheric and bulk) and plants (aerial and subterranean parts) were refrigerated and processed within 24 h after sampling.

### 4.2. Induced Respiration (IR)

The respiration analysis was carried out using a MicroResp^®^ microplate-based system, according to the manufacturer’s manual [27,76], with modifications. Following manual recommendations and availability, multiple carbon sources were used as substrates for IR: citric acid (CiAc), malic acid (MaAc), galactose (Gal), and glucose (Glu). Absorbance readings were made at 595 nm and converted into respiration rates (µg CO_2_ released by g soil^−1^ h^−1^). For CO_2_ calculations, each microplate included a row of calibration wells, in which known amounts of inorganic Rx citric acid + sodium bicarbonate were added to produce known amounts of sodium citrate + carbon dioxide. The absorbance readings of the calibration wells in the plate were transformed into CO_2_ liberation rates (respiration) thanks to a calibration curve of CO_2_ liberation, with the same reaction created with Dansensor’s Checkpoint O_2_/CO_2_^®^.

### 4.3. Enzymatic Activities (EA) Analysis

Acid and alkaline phosphomonoesterase (AcPh and AlPh, respectively) activity were measured based on the method of Tabatabai and Bermner [77] (phosphatases activity as µmol p-nitrophenol soil g^−1^ h^−1^). It consisted of the spectrophotometric determination of the p-nitrophenol released when the soil is incubated at 37 °C for 1 h with a buffered solution (pH = 6.5 for acid, pH = 11 for alkaline) of p-nitrophenylphosphate. The amount of released p-nitrophenol was measured with a spectrophotometer at a 400 nm wavelength. To obtain the final concentrations, the raw absorbance data by sample were interpolated in the calibration of the standard curve.

B-glucosidase (BGlu) activity was measured as described in Tabatabai [78]. The colorimetric method is based on the determination of the p-nitrophenol obtained by the action of the enzyme β-glucosidase after incubating the soil with the substrate p-nitrophenol β-D-glucopyranoside at pH 6. The incubation was carried out at 37 °C for an hour, and the released p-nitrophenol was removed by filtration after the addition of CaCl_2_ and THAM-NaOH pH 12. With the absorbance readings at 400 nm of the standard, the calibration curve was calculated and used to obtain the concentration in µmol p-nitrophenol soil g^−1^ h^−1^. With the values obtained, net activity was calculated.

Dehydrogenase (DeHy) activity was measured as described in Trevors [79] and García et al. [80]. The enzymatic reaction is based on the spectrophotometric measurement at the wavelength of 540 nm of the iodonitrotetrazolium formazan (INTF), formed when the soil is incubated with 2-p-iodophenyl-3-p-nitrophenyl-5-phenyltetrazolium (INT) in the dark for 20 h at 20 °C. The absorbance for the sample was interpolated into the equation of the calibration curve, obtaining the amount of µmol INTF soil g^−1^ h^−1^.

Urease (Ur) activity was measured according to Kandeler and Gerber [81] and modified by Kandeler et al. [82]. This colorimetric method is based on the determination of the ammonium released in the incubation of a soil solution at 37 °C for 2 h, where the ammonia produced by urease activity reacts with salicylate and dichloro isocyanide to give a bluish-green color. The absorbance at 610 nm was converted to the ammonia nitrogen concentration (µmol N-NH_4_^+^ soil g^−1^ h^−1^).

### 4.4. Microbial Count (MC)

One gram of soil per sample was used to cultivate and count different soil microorganisms: total viable aerobic mesophilic bacteria count (TVA) was made with Plate Count Agar (Scharlau, Barcelona, Spain) at 28 °C for 48 h, total molds and yeast count (TMY) with Saboureaud Chloramphenicol Agar (Scharlau, Barcelona, Spain), at 28 °C for 3 d, total strictly anaerobic bacteria count (Tan) with Schaendler Agar (Conda Pronadisa, Madrid, Spain) at 37 °C in CO_2_ atmosphere for 48 h, and total oligotrophic bacteria count (TO) with Oligotrophic Agar, composed of dipotassium phosphate (Panreac, Barcelona, Spain), magnesium sulfate heptahydrate (Panreac, Barcelona, Spain), peptone (Scharlau, Barcelona, Spain), glycerin (Scharlau, Barcelona, Spain), and bacteriologic agar (Labkem, Dublin, Ireland) [83] at 28 °C, 5 d. All the analyses were performed in duplicate. Results were expressed in log CFU/g of soil.

### 4.5. N_2_ Cycle (NC)

Rhizosphere soil samples were tested for their capacity to perform different steps of the N_2_ cycle. To evaluate the mineralization, nitrification I and II, and denitrification potential of the rhizospheres, 0.1 g of soil was added to different substrates (as follows) and incubated at 28 °C for 1 to 3 weeks. Then, measurements were performed each week of the NH_4^+^_, NO_2^−^_, NO_3^−^_ and N_2_ production, respectively.

Peptone water (Scharlau, Barcelona, Spain) was used as the substrate for the mineralization process (Am). NH_4_^+^ production was measured seven days after sowing using a colorimetric test strip method based on the Neßler reagent (Mquant TM Ammonium Test, Merk Darmstadt, Germany).

Ammonium sulfate solution was used to measure the conversion of ammonia into nitrate, from now on called nitrification I (NitI), after 14 days of incubation using test strips (Mquant TM, Nitrites test Merck Darmstadt, Germany). The concentration of nitrite ions was calculated by observing the color change produced by a reddish violet azodye, which is formed due to the diazotization of the nitrosating species and subsequent coupling with N-(1-naphthyl) ethylenediamine [84].

Nitrite solution was used to measure nitrification II with the same frequency as the previous one by means of test strips (Mquant TM Nitrates Test Merk Darmstadt, Germany). Nitrate ions are measured based on the same chemical reactions as in nitrification I. The nitrate ion is reduced to nitrite ion; these nitrite ions, in an acidic medium, form the nitrosating species and, when reacting with an aromatic amine, undergo diazotization, forming a diazonium salt. Later, it binds to N-(1-naphthyl) ethylenediamine, giving rise to a reddish violet azodye [84].

Nitrate solution was used to measure denitrification through the level of nitrogen gas produced at 7 days, 14 days, and 21 days, and was assessed with a Durham hood gas production expressed in a range from 0 to 4. The results were expressed in mg NH_4^+^_-N/g, mg NO_2^−^_-N/g, mg NO_3^−^_-N/g, and N_2_ production in a 0–4 scale.

### 4.6. Evaluation of Plant Samples

Plant biomass (shoots, fruits, and roots parts) was weighted fresh and dry. All parameters were sampled at PS-1 and PS-2, except stump and fruit weight. These were only obtained at PS-2, as the plants were not big enough at PS-1, and the stump was not very differentiated and had no fruits: aerial part fresh weight (AeFW), aerial part dry weight (AeDW), fruits fresh weight (FFW), root fresh weight (RFW), root dry weight (RDW), stump fresh weight (SFW), and stump dry weight (SDW).

Roots were washed, spread in a transparent sheet, and scanned to be measured. Images of scanned roots were measured with WinRHIZO-Pro 2003b, obtaining: average diameter (AD, cm), branching density (BD, number of forks by total length), number of forks (NF), root density (RD, dry weight (g) by volume (cm^3^)), surface area (SA, cm^2^), total length (TL, cm), total volume (TV, m^3^), and length by diameter classes (0.5, 0.5–2.5, and >2.5, mm).

### 4.7. Statistical Analysis

To determine the differences between the main effects (accessions and bulk soil, FS, and interaction) and multivariate analysis of variance (MANOVA) for every trait, Student–Newman–Keuls (SNK) tests were carried out to compare accessions at each farming system and time using Statgraphics software V.18.1.13 (64-bits).

The rest of the analyses were carried out with R (R Core Team, Viena, Austria, ×64 V. 4.1.0 (18 May 2021) in Rstudio Team, V. 1.4.1106 (11 February 2021)): Pearson’s multiple correlation coefficient analysis was carried out for each stage (full heatmap in Supplementary Data), and the packages used were: corrplot [85], svglite [86]. Exploratory factor analysis (EFA) was carried out at each phenological stage. For EFA, packages used were psych [87], tidyverse [88], mvnormtest [89], nFactors [90], EFA.MRFA [91], and dplyr [92]. Final numbers of factors were decided as a three-criteria decision-making path: number of eigenvalues greater than one, parallel analysis, and previous knowledge of the main effects (MANOVA’s effects).

## 5. Conclusions

The present study contributes novel insights into how pepper genotypes, farming systems, and soils interact. Clear differences in the bulk and rhizosphere soils depending on the farming system indicate that it is a key factor in shaping the health status of soil, nutrient cycling capacity, and emissions. However, we are still far from understanding all possible changes and their consequences for food production and the global environment. Pepper genotypes changed the rhizosphere’s functionality in a specific way, probably because of specific exudates. However, again, future studies are needed to discover those exudate profiles, their relationship with the proliferation of certain microorganisms, and their relationship with the root architecture. A combined study on plant genotypes and soil microbial profiles should be conducted in the future to create soil-tailored sustainable agriculture.

## Figures and Tables

**Table 1:** Mean values (n = 3) of substrate induced respiration (µg CO_2_ g soil^−1^ h^−1^) from rhizospheres and bulk soil samples using different substrates: citric acid (CiAc), malic acid (MaAc), galactose (Gal), glucose (Glu), in two farming systems (organic; org and conventional; con) at two sampling times (T1: summer, T2: autumn).

Sample	Substrate	Farming System	*BOL-58*	*Serrano*	*Bola*	*Piquillo*	Bulk	Average
T1	CiAc	Org	08.35 ^a^	08.09 ^a^	45.82 ^b^	15.58 ^a^	10.65 ^a^	17.70
		Con	60.92 ^ns^	06.17 ^ns^	38.19 ^ns^	82.02 ^ns^	77.80 ^ns^	53.02 *
	MaAc	Org	08.86 ^a^	08.88 ^a^	46.58 ^b^	18.66 ^a^	24.35 ^a^	21.47
		Con	70.90 ^ns^	13.51 ^ns^	43.07 ^ns^	61.08 ^ns^	46.54 ^ns^	47.02 *
	Gal	Org	01.69 ^ns^	01.66 ^ns^	01.89 ^ns^	01.36 ^ns^	01.29 ^ns^	01.58
		Con	02.75 ^ns^	01.07 ^ns^	04.02 ^ns^	02.91 ^ns^	04.06 ^ns^	02.96 *
	Glu	Org	03.68 ^ns^	04.33 ^ns^	04.00 ^ns^	03.92 ^ns^	04.16 ^ns^	04.02
		Con.	06.14 ^ns^	02.46 ^ns^	12.58 ^ns^	07.67 ^ns^	40.16 ^ns^	13.80 *
T2	CiAc	Org.	25.75 ^ab^	58.76 ^b^	37.33 ^ab^	08.13 ^a^	12.24 ^a^	28.44 ^NS^
		Con.	22.62 ^ns^	51.84 ^ns^	42.64 ^ns^	35.99 ^ns^	06.87 ^ns^	31.99 ^NS^
	MaAc	Org.	29.80 ^ns^	43.62 ^ns^	50.53 ^ns^	08.06 ^ns^	05.51 ^ns^	27.50 ^NS^
		Con.	27.98 ^ns^	30.28 ^ns^	43.57 ^ns^	32.39 ^ns^	22.37 ^ns^	31.32 ^NS^
	Gal	Org.	01.81 ^ns^	01.80 ^ns^	02.22 ^ns^	01.64 ^ns^	01.66 ^ns^	01.83 *
		Con.	01.13 ^ab^	02.08 ^b^	01.66 ^ab^	00.41 ^a^	00.47 ^a^	01.15
	Glu	Org.	04.01 ^ns^	05.87 ^ns^	06.56 ^ns^	03.41 ^ns^	03.82 ^ns^	04.73 *
		Con.	01.69 ^ns^	02.39 ^ns^	01.77 ^ns^	02.12 ^ns^	05.10 ^ns^	02.61

* Mean values with different lower-case letters within rows indicate significant differences among rhizospheres including bulk soil based on the Student–Newman–Keuls multiple range test at *p*-value < 0.05, ns indicates no significant differences. Asterisk in last column (average), indicates significant differences between farming systems for pairs of data from the same substrate at *p*-value < 0.05, NS in the last column indicates no significant differences between farming systems for pairs of data from the same substrate.

**Table 2:** Mean values (n = 3) of enzymatic activity of the rhizospheres and the bulk soil for acid phosphatase (AcPh, µmol p-nitrophenol soil g^−1^ h^−1^), alkaline phosphatase (AlPh, µmol p-nitrophenol soil g^−1^ h^−1^), β-glucosidase (βGlu, µmol p-nitrophenol soil g^−1^ h^−1^), dehydrogenase (DeHy, µmol INTF soil g^−1^ h^−1^), and urease (Ur, µmol N-NH_4_^+^ soil g^−1^ h^−1^) in two farming systems (organic: org or conventional: con) at two sampling times (T1, summer and T2, autumn).

Sampling	Enzyme	Farming System	*BOL-58*	*Serrano*	*Bola*	*Piquillo*	Bulk	Average
T1	AcPh	Org.	90.16 ^ns^	89.34 ^ns^	98.49 ^ns^	87.59 ^ns^	89.85 ^ns^	91.09 *
		Con.	76.15 ^ns^	65.68 ^ns^	76.81 ^ns^	80.23 ^ns^	70.56 ^ns^	73.89
	AlPh	Org.	455.69 ^a^	563.92 ^b^	450.40 ^a^	429.01 ^a^	467.92 ^a^	473.39 ^NS^
		Con.	460.04 ^ns^	402.87 ^ns^	435.10 ^ns^	459.57 ^ns^	526.41 ^ns^	456.80 ^NS^
	βGlu	Org.	00.72 ^ns^	00.61 ^ns^	00.80 ^ns^	00.68 ^ns^	00.79 ^ns^	00.72 *
		Con.	00.40 ^ns^	00.60 ^ns^	00.55 ^ns^	00.44 ^ns^	00.39 ^ns^	00.48
	DeHy	Org.	01.18 ^b^	00.95 ^a^	00.91 ^a^	00.99 ^a^	00.94 ^a^	00.99 ^NS^
		Con.	01.08 ^b^	00.78 ^a^	01.14 ^b^	01.07 ^b^	01.02 ^b^	01.02 ^NS^
	Ur	Org.	00.16 ^ns^	00.17 ^ns^	00.17 ^ns^	00.18 ^ns^	00.20 ^ns^	00.18 ^NS^
		Con.	00.14 ^ns^	00.13 ^ns^	00.15 ^ns^	00.20 ^ns^	00.14 ^ns^	00.15 ^NS^
T2	AcPh	Org.	93.99 ^ns^	85.00 ^ns^	95.04 ^ns^	87.75 ^ns^	87.60 ^ns^	89.88 *
		Con.	76.11 ^ns^	69.38 ^ns^	72.79 ^ns^	76.03 ^ns^	78.22 ^ns^	74.51
	AlPh	Org.	477.13 ^a^	569.41 ^b^	472.26 ^a^	449.11 ^a^	458.76 ^a^	485.33 *
		Con.	406.98 ^ns^	350.58 ^ns^	420.99 ^ns^	447.58 ^ns^	510.30 ^ns^	427.29
	βGlu	Org.	00.62 ^ns^	00.70 ^ns^	00.67 ^ns^	00.60 ^ns^	00.52 ^ns^	00.62 *
		Con.	00.53 ^ns^	00.43 ^ns^	00.50 ^ns^	00.44 ^ns^	00.34 ^ns^	00.45
	DeHy	Org.	01.04 ^ns^	00.94 ^ns^	00.96 ^ns^	00.99 ^ns^	00.98 ^ns^	00.98 ^NS^
		Con.	01.02 ^ns^	01.03 ^ns^	01.14 ^ns^	00.99 ^ns^	01.07 ^ns^	01.05 ^NS^
	Ur	Org.	00.31 ^a^	00.36 ^a^	00.47 ^b^	00.35 ^a^	00.30 ^a^	00.36 *
		Con.	00.23 ^ns^	00.19 ^ns^	00.19 ^ns^	00.20 ^ns^	00.18 ^ns^	00.20

* Mean values with different lower-case letters within rows indicate significant differences among rhizospheres including bulk soil based on the Student–Newman–Keuls multiple range test at *p*-value < 0.05, whereas, ns indicates no significant differences. Asterisk in last column (average), indicates significant differences between farming systems for pairs of data from the same substrate at *p*-value < 0.05, NS in the last column indicates no significant differences between farming systems for pairs of data from the same substrate.

**Table 3:** Mean values of microbial counts (log CFU g soil^−1^) from rhizospheres and bulk soil samples using different growing media: total viable aerobic mesophilic bacterial count (TVA), total molds and yeast count (TMY), total anaerobic bacteria (TAn), total oligotrophic bacterial count (TO) in two farming systems (organic; org and conventional; con) at two sampling times (T1: summer, T2: autumn).

Sampling	Microbial Count	Farming System	*BOL-58*	*Serrano*	*Bola*	*Piquillo*	Bulk	Average
T1	TVA	Org	7.34 ^ns^	7.51 ^ns^	7.53 ^ns^	7.37 ^ns^	7.27 ^ns^	7.40 *
		Con	6.88 ^ns^	7.00 ^ns^	6.78 ^ns^	7.04 ^ns^	6.89 ^ns^	6.92
	TMY	Org	8.30 ^ns^	4.67 ^ns^	5.57 ^ns^	5.12 ^ns^	4.45 ^ns^	5.62 ^NS^
		Con	5.64 ^ns^	5.78 ^ns^	5.59 ^ns^	5.06 ^ns^	5.40 ^ns^	5.50 ^NS^
	TAn	Org	7.29 ^ns^	5.98 ^ns^	6.67 ^ns^	6.57 ^ns^	5.74 ^ns^	6.45 *
		Con	5.64 ^ns^	5.69 ^ns^	5.53 ^ns^	5.90 ^ns^	5.39 ^ns^	5.63
	TO	Org	7.18 ^ns^	7.33 ^ns^	7.55 ^ns^	7.33 ^ns^	7.23 ^ns^	7.33 ^NS^
		Con.	7.18 ^ns^	7.49 ^ns^	7.38 ^ns^	6.76 ^ns^	7.24 ^ns^	7.21 ^NS^
T2	TVA	Org	7.62 ^ns^	7.40 ^ns^	7.43 ^ns^	7.46 ^ns^	7.36 ^ns^	7.45 *
		Con	6.19 ^a^	7.79 ^b^	7.05 ^ab^	6.42 ^a^	7.01 ^ab^	6.89
	TMY	Org	4.76 ^ns^	5.47 ^ns^	5.43 ^ns^	5.33 ^ns^	5.44 ^ns^	5.329 ^NS^
		Con	5.10 ^ns^	5.32 ^ns^	5.11 ^ns^	5.05 ^ns^	4.97 ^ns^	5.11 ^NS^
	TAn	Org	5.81 ^ns^	5.66 ^ns^	6.18 ^ns^	5.82 ^ns^	5.68 ^ns^	5.83 ^NS^
		Con	5.42 ^ns^	6.86 ^ns^	6.13 ^ns^	5.68 ^ns^	5.94 ^ns^	6.01 ^NS^
	TO	Org	7.41 ^ns^	7.29 ^ns^	7.19 ^ns^	7.31 ^ns^	7.22 ^ns^	7.28 *
		Con.	6.73 ^ns^	6.58 ^ns^	6.85 ^ns^	6.64 ^ns^	6.48 ^ns^	6.66

* Mean values with different lower-case letters within rows indicate significant differences among rhizospheres including bulk soil based on the Student–Newman–Keuls multiple range test at *p*-value < 0.05, ns indicates no significant differences. Asterisk in last column (average), indicates significant differences between farming systems for pairs of data from the same substrate at *p*-value < 0.05, NS in the last column indicates no significant differences between farming systems for pairs of data from the same substrate.

**Table 4:** Mean values of nitrogen catabolism products of the rhizospheres and the bulk soil for ammonification potential (Am, mg NH_4_^+^-N g^−1^), nitrification I potential (NIT-I, mg NO_2_^+^-N g^−1^), nitrification II potential (NIT-II, mg NO_3_^+^-N g^−1^), denitrification potential (DN, 0–4 scale) in two farming systems (organic: org or conventional: con) at two sampling times (T1, summer and T2, autumn).

Sampling	N-Cycle Stage	Farming System	*BOL-58*	*Serrano*	*Bola*	*Piquillo*	Bulk	Average
T1	Am	Org	232.8 ^ns^	119 ^ns^	206.9 ^ns^	144.8 ^ns^	75.0 ^ns^	155.7 ^NS^
		Con	100.9 ^a^	119 ^a^	31.0 ^a^	310.4 ^b^	51.7 ^a^	122.6 ^NS^
	NIT-I	Org	24.32 ^ns^	16.21 ^ns^	20.27 ^ns^	20.27 ^ns^	20.27 ^ns^	20.27 *
		Con	2.28 ^ns^	3.24 ^ns^	1.72 ^ns^	2.03 ^ns^	3.55 ^ns^	2.56
	NIT-II	Org	22.60 ^ns^	15.07 ^ns^	18.83 ^ns^	18.83 ^ns^	18.83 ^ns^	18.83 ^NS^
		Con	22.60 ^ns^	22.60 ^ns^	18.83 ^ns^	22.60 ^ns^	22.60 ^ns^	21.85 ^NS^
	DN	Org	2.50 ^ns^	3.33 ^ns^	1.00 ^ns^	2.33 ^ns^	3.67 ^ns^	2.57 ^NS^
		Con.	3.50 ^ns^	2.67 ^ns^	3.33 ^ns^	3.33 ^ns^	2.33 ^ns^	3.03 ^NS^
T2	Am	Org.	77.60 ^ns^	38.80 ^ns^	77.60 ^ns^	67.25 ^ns^	103.5 ^ns^	72.94 *
		Con.	6.73 ^a^	3.10 ^a^	4.91 ^a^	31.04 ^b^	15.52 ^a^	12.26
	NIT-I	Org.	0.20 ^a^	0.76 ^a^	2.53 ^b^	0.20 ^a^	0.20 ^a^	0.78 *
		Con.	0.15 ^ns^	0.22 ^ns^	0.15 ^ns^	0.30 ^ns^	0.35 ^ns^	0.24
	NIT-II	Org.	18.83 ^ns^	16.95 ^ns^	18.83 ^ns^	18.83 ^ns^	22.60 ^ns^	19.21 *
		Con.	1.51 ^ns^	1.88 ^ns^	2.26 ^ns^	2.26 ^ns^	2.26 ^ns^	2.03
	DN	Org.	1.33 ^ns^	2.50 ^ns^	1.67 ^ns^	1.00 ^ns^	2.33 ^ns^	1.77
		Con.	3.00 ^ns^	2.00 ^ns^	3.67 ^ns^	3.67 ^ns^	2.67 ^ns^	3.00 *

* Mean values with different lower-case letters within rows indicate significant differences among rhizospheres including bulk soil based on the Student–Newman–Keuls multiple range test at *p*-value < 0.05, ns indicates no significant differences. Asterisk in last column (average) indicates significant differences between farming systems for pairs of data from the same substrate at *p*-value < 0.05, NS in the last column indicates no significant differences between farming systems for pairs of data from the same substrate.

**Table 5:** Mean values (n = 3) of biomass and yield traits (g) from accessions: Aerial dry weight (AeDW), aerial fresh weight (AeFW), root dry weight (RDW), root fresh weight (RFW), fruit fresh weight (FFW), stump dry weight (SDW), stump fresh weight (SFW) in two farming systems (organic; org and conventional; con) at two sampling times (T1: summer, T2: autumn).

Sample	Biomass Traits	Farming System	*BOL-58*	*Serrano*	*Bola*	*Piquillo*	Average
T1	AeDW	Org	15.40 ^ns^	14.74 ^ns^	9.73 ^ns^	8.69 ^ns^	12.14 ^NS^
		Con	5.97 ^ns^	7.95 ^ns^	17.27 ^ns^	15.91 ^ns^	11.78 ^NS^
	AeFW	Org	106.15 ^ns^	84.86 ^ns^	56.36 ^ns^	49.56 ^ns^	74.23 ^NS^
		Con	44.85 ^ns^	48.75 ^ns^	118.65 ^ns^	116.83 ^ns^	82.27 ^NS^
	RDW	Org	1.79 ^ns^	1.98 ^ns^	1.35 ^ns^	1.22 ^ns^	1.58 ^NS^
		Org	0.83 ^a^	0.69 ^a^	2.24 ^b^	1.85 ^b^	1.40 ^NS^
	RFW	Org	7.98 ^ns^	10.72 ^ns^	6.94 ^ns^	5.48 ^ns^	7.78
		Con	4.90 ^a^	4.84 ^a^	21.49 ^b^	15.63 ^b^	11.72 *
T2	AeDW	Org	239.67 ^ns^	329.00 ^ns^	117.67 ^ns^	175.00 ^ns^	215.3 ^NS^
		Con	369.33 ^b^	262.00 ^ab^	112.33 ^a^	122.67 ^a^	216.6 ^NS^
	AeFW	Org	1103.7 ^ns^	1173.0 ^ns^	407.00 ^ns^	723.33 ^ns^	851.7 ^NS^
		Con	1533.00 ^b^	883.00 ^ab^	471.18 ^a^	574.57 ^a^	865.4 ^NS^
	RDW	Org	7.60 ^ns^	15.06 ^ns^	3.45 ^ns^	12.13 ^ns^	9.56 *
		Con	6.30 ^b^	10.66 ^c^	2.62 ^a^	5.68 ^b^	6.32
	RFW	Org	19.91 ^ab^	33.69 ^ab^	13.43 ^a^	43.68 ^b^	27.68 *
		Con	14.83 ^ab^	23.90 ^b^	9.64 ^a^	18.86 ^ab^	16.80
	FFW	Org	557.00 ^ns^	1849.0 ^ns^	419.33 ^ns^	714.67 ^ns^	885.0
		Con	1047.4 ^ns^	1629.5 ^ns^	1261.0 ^ns^	1657.3 ^ns^	1398.8 *
	SDW	Org	17.61 ^ns^	25.10 ^ns^	10.89 ^ns^	19.45 ^ns^	18.26 ^NS^
		Con	16.48 ^ns^	26.41 ^ns^	15.24 ^ns^	19.84 ^ns^	19.49 ^NS^
	SFW	Org	47.94 ^ns^	69.60 ^ns^	35.82 ^ns^	58.58 ^ns^	52.99 ^NS^
		Con	44.72 ^ns^	65.79 ^ns^	52.14 ^ns^	61.64 ^ns^	56.08 ^NS^

* Mean values with different lower-case letters within rows indicate significant differences among rhizospheres including bulk soil based on the Student–Newman–Keuls multiple range test at *p*-value < 0.05, ns indicates no significant differences. Asterisk in last column (average) indicates significant differences between farming systems for pairs of data from the same substrate at *p*-value < 0.05, NS in the last column indicates no significant differences between farming systems for pairs of data from the same substrate.

**Table 6:** Mean values (n = 3) of root measurements from accessions: Average root diameter (AD, mm), branching density as number of total forks by length (BD, cm^−1^), number of forks (NF, number), root density (RD, g cm^−3^), surface area (SA, cm^2^), total length (TL, cm), total volume (TV, cm^3^), total length of roots with diameter less than 0.5 mm (<0.5, cm), total length of roots with diameter between 0.5 mm and 2.5 mm (0.5–2.5, cm), total length of roots with diameter higher than 2.5 mm (>2.5, mm) in two farming systems (organic; org and conventional; con) at two sampling times (T1: summer, T2: autumn).

Sample	Root Traits	Farming System	*BOL-58*	*Serrano*	*Bola*	*Piquillo*	Average
T1	AD	Org	0.88 ^a^	1.06 ^b^	0.77 ^a^	0.80 ^a^	0.88 ^NS^
		Con	0.75 ^ns^	0.79 ^ns^	0.88 ^ns^	0.96 ^ns^	0.85 ^NS^
	BD	Org	01.08 ^ns^	00.84 ^ns^	00.93 ^ns^	00.94 ^ns^	00.95 *
		Con	00.90 ^b^	00.76 ^ab^	00.59 ^a^	00.74 ^ab^	00.75
	NF	Org	896.50 ^ns^	539.00 ^ns^	1001.00 ^ns^	715.33 ^ns^	787.96 ^NS^
		Con	987.00 ^ab^	588.00 ^a^	1375.67 ^b^	1230.67 ^b^	1045.33 ^NS^
	RD	Org	0.36 ^ns^	0.35 ^ns^	0.30 ^ns^	0.31 ^ns^	0.33 *
		Con	0.16 ^ns^	0.17 ^ns^	0.15 ^ns^	0.16 ^ns^	0.16
	SA	Org	229.96 ^ns^	214.40 ^ns^	249.69 ^ns^	182.88 ^ns^	219.23
		Con	263.43 ^a^	190.60 ^a^	658.16 ^b^	499.66 ^b^	402.96 *
	TL	Org	827.97 ^ns^	634.98 ^ns^	1042.61 ^ns^	728.04 ^ns^	808.40
		Con	1097.45 ^a^	767.85 ^a^	2356.56 ^c^	1650.75 ^b^	1468.15 *
	TV	Org	5.09 ^ns^	5.85 ^ns^	4.76 ^ns^	3.70 ^ns^	4.85
		Con	5.15 ^a^	3.80 ^a^	14.91 ^b^	12.20 ^b^	9.01 *
	<0.5	Org.	411.63 ^ns^	258.57 ^ns^	558.74 ^ns^	353.67 ^ns^	395.65
		Con.	550.34 ^ab^	386.05 ^a^	732.84 ^b^	550.65 ^ab^	554.97 *
	0.5–2.5	Org.	403.25 ^ns^	350.15 ^ns^	473.91 ^ns^	365.93 ^ns^	398.31
		Con.	539.32 ^a^	374.29 ^a^	1608.41 ^c^	1088.20 ^b^	902.56 *
	>2.5	Org.	12.43 ^a^	25.11 ^b^	07.62 ^a^	06.78 ^a^	12.98 *
		Con.	06.43 ^a^	07.32 ^a^	13.29 ^b^	10.79 ^ab^	09.45
T2	AD	Org	0.77 ^a^	0.99 ^b^	0.69 ^a^	0.81 ^a^	0.82
		Con	0.93 ^ns^	1.06 ^ns^	0.93 ^ns^	0.83 ^ns^	0.94 *
	BD	Org	01.84 ^b^	01.15 ^a^	01.63 ^b^	01.60 ^b^	01.56 *
		Con	01.60 ^b^	00.87 ^a^	01.01 ^a^	01.14 ^a^	01.15
	NF	Org	5567.33 ^a^	2408.50 ^a^	4940.33 ^a^	9037.67 ^b^	5488.46 *
		Con	3913.67 ^ns^	1373.50 ^ns^	1759.33 ^ns^	2862.00 ^ns^	2477.13
	RD	Org	0.50 ^a^	0.88 ^b^	0.28 ^a^	0.40 ^a^	0.52 ^NS^
		Con	0.40 ^a^	0.74 ^b^	0.29 ^a^	0.40 ^a^	0.45 ^NS^
	SA	Org	742.55 ^a^	645.90 ^a^	668.90 ^a^	1450.86 ^b^	877.05 *
		Con	695.48 ^ns^	506.02 ^ns^	465.67 ^ns^	646.71 ^ns^	578.47
	TL	Org	2976.85 ^a^	2102.00 ^a^	3034.65 ^a^	5622.49 ^b^	3434.00 *
		Con	2449.62 ^ns^	1554.31 ^ns^	1592.53 ^ns^	2452.94 ^ns^	2012.35
	TV	Org	14.88 ^a^	16.51 ^a^	12.17 ^a^	30.60 ^b^	18.54 ^NS^
		Con	22.23 ^ns^	14.40 ^ns^	10.97 ^ns^	14.62 ^ns^	15.56 ^NS^
	<0.5	Org.	1860.15 ^a^	1212.32 ^a^	1935.92 ^a^	3267.68 ^b^	2069.02 *
		Con.	1378.57 ^ns^	841.44 ^ns^	702.66 ^ns^	1426.58 ^ns^	1087.31
	0.5–2.5	Org.	1006.41 ^a^	771.91 ^a^	1020.79 ^a^	2151.75 ^b^	1237.71 *
		Con.	997.92 ^ns^	580.75 ^ns^	839.86 ^ns^	937.97 ^ns^	839.12
	>2.5	Org.	107.48 ^ab^	117.57 ^ab^	74.77 ^a^	198.18 ^b^	124.50 *
		Con.	69.95 ^ab^	129.53 ^b^	48.82 ^a^	85.88 ^ab^	83.55

* Mean values with different lower-case letters within rows indicate significant differences among rhizospheres including bulk soil based on the Student–Newman–Keuls multiple range test at *p*-value < 0.05, ns indicates no significant differences. Asterisk in last column (average) indicates significant differences between farming systems for pairs of data from the same substrate at *p*-value < 0.05, NS in the last column indicates no significant differences between farming systems for pairs of data from the same substrate.

**Table 7:** Factor loads for rhizosphere and bulk soil analyzed parameters at two sampling times. Citric acid (CiAc), malic acid (MaAc), galactose (Gal), glucose (Glu), acid phosphatase (AcPh), β-glucosidase (βGlu), dehydrogenase (DeHy), and urease (Ur), total viable aerobic mesophilic bacterial count (TVA), total molds and yeast count (TMY), total anaerobic bacteria (TAn), total oligotrophic bacterial count (TO), ammonification potential (Am), nitrification I potential (NIT-I), nitrification II potential (NIT-II), denitrification potential (DN) in two farming systems (organic: org or conventional: con) at two sampling times (T1, summer and T2, autumn).

	T1			T2		
Trait	F1.1	F2.1	F3.1	F1.2	F2.2	F3.2
CiAc	0.83	−0.36	0.19	−0.04	0.91	0.16
MaAc	0.57	−0.38	−0.03	0.01	0.92	−0.03
Gal	0.77	0.14	−0.06	0.45	0.26	0.55
Glu	0.71	0.12	−0.17	0.50	0.22	0.09
AcPh	−0.35	0.19	0.66	0.60	−0.06	0.07
βGlu	−0.50	0.29	0.40	0.73	0.33	0.12
DeHy	-	-	-	−0.40	0.06	0.19
Ur	−0.05	−0.04	0.72	0.97	0.10	0.06
TVA	−0.15	0.73	0.37	0.27	−0.03	0.91
TMY	0.12	0.43	0.32	0.24	0.18	0.14
TAn	−0.25	0.47	0.32	−0.17	0.19	0.45
TO	0.05	0.81	−0.16	0.81	−0.08	0.28
Am	0.06	0.01	0.51	0.68	−0.26	0.24
NIT-I	−0.57	0.29	0.37	0.44	0.28	0.03
NIT-II	0.13	−0.14	−0.42	0.87	−0.19	0.26
DN	0.05	−0.16	−0.01	−0.39	0.20	−0.48
% of Var.	30.21	14.38	12.26	35.89	15.46	10.67
Eigenvalues	4.53	2.16	1.84	5.74	2.47	1.71

## Data Availability

R scripts and data used for statistical analysis and figures are available at: https://github.com/ivalethia/efa-root-soil, (30 April 2022).

## Supplementary Materials

The following supporting information can be downloaded at: https://www.mdpi.com/article/10.3390/plants12091873/s1, Table S1: Scorings of accessions, bulks, and trait loads; Table S2: Monthly temperatures and precipitation.

## References

[1] Rodriguez-Burruezo A., González-Mas M.D.C., Nuez F. (2010). Carotenoid composition and vitamin A value in ají (*Capsicum baccatum* L.) and rocoto (*C. pubescens* R. & P.), 2 pepper species from the Andean region. J. Food Sci..

[2] FAOSTAT Statistic Division, Food and Agriculture Organization of the United Nations. http://www.fao.org/faostat/.

[3] (2021). MAPA Ministerio de Agricultura, Pesca y Alimentación. https://www.mapa.gob.es/es/.

[4] Willer H., Trávníček J., Meier C., Schlatter B. (2021). The World of Organic Agriculture 2021-Statistics and Emerging Trends.

[5] Ribes-Moya A.M., Raigon M.D., Moreno-Peris E., Fita A., Rodriguez-Burruezo A. (2018). Response to organic cultivation of heirloom Capsicum peppers: Variation in the level of bioactive compounds and effect of ripening. PLoS ONE.

[6] Ribes-Moya A.M., Adalid A.M., Raigon M.D., Hellín P., Fita A., Rodriguez-Burruezo A. (2020). Variation in flavonoids in a collection of peppers (*Capsicum* sp.) under organic and conventional cultivation: Effect of the genotype, ripening stage, and growing system. J. Sci. Food Agric..

[7] Tripodi P., Cardi T., Bianchi G., Migliori C.A., Schiavi M., Rotino G.L., Lo Scalzo R. (2018). Genetic and environmental factors underlying variation in yield performance and bioactive compound content of hot pepper varieties (*Capsicum annuum*) cultivated in two contrasting Italian locations. Eur. Food Res. Technol..

[8] Zheng Q., Hu Y., Zhang S., Noll L., Böckle T., Dietrich M., Herbold C.W., Eichorst S.A., Woebken D., Richter A. (2019). Soil multifunctionality is affected by the soil environment and by microbial community composition and diversity. Soil Biol. Biochem..

[9] Yang J., Kloepper J.W., Ryu C.M. (2009). Rhizosphere bacteria help plants tolerate abiotic stress. Trends Plant Sci..

[10] Kandasamy S., Loganathan K., Muthuraj R., Duraisamy S., Seetharaman S., Thiruvengadam R., Ponnusamy B., Ramasamy S. (2009). Understanding the molecular basis of plant growth promotional effect of *Pseudomonas fluorescens* on rice through protein profiling. Proteome Sci..

[11] Kloepper J.W., Ryu C.M., Zhang S. (2004). Induced systemic resistance and promotion of plant growth by *Bacillus* spp.. Phytopathology.

[12] Singh B.K., Millard P., Whiteley A.S., Murrell J.C. (2004). Unravelling rhizosphere–microbial interactions: Opportunities and limitations. Trends Microbiol..

[13] Wang X., Whalley W.R., Miller A.J., White P.J., Zhang F., Shen J. (2020). Sustainable Cropping Requires Adaptation to a Heterogeneous Rhizosphere. Trends Plant Sci..

[14] Pereira-Dias L., Gil-Villar D., Castell-Zeising V., Quiñones A., Calatayud Á., Rodríguez-Burruezo A., Fita A. (2020). Main root adaptations in pepper germplasm (*Capsicum* spp.) to phosphorus low-input conditions. Agronomy.

[15] Sánchez-Cañizares C., Jorrín B., Poole P.S., Tkacz A. (2017). Understanding the holobiont: The interdependence of plants and their microbiome. Curr. Opin. Microbiol..

[16] Aira M., Gomez-Brandon M., Lazcano C., Bååth E., Dominguez J. (2010). Plant genotype strongly modifies the structure and growth of maize rhizosphere microbial communities. Soil Biol. Biochem..

[17] Toljander J.F., Lindahl B.D., Paul L.R., Elfstrand M., Finlay R.D. (2007). Influence of arbuscular mycorrhizal mycelial exudates on soil bacterial growth and community structure. FEMS Microbiol. Ecol..

[18] Edwards C.A. (2004). Earthworm Ecology.

[19] Tate R.L. (2000). Soil Microbiology.

[20] Nelkner J., Henke C., Lin T.W., Pätzold W., Hassa J., Jaenicke S., Grosch R., Pühler A., Sczyrba A., Schlüter A. (2019). Effect of Long-Term Farming Practices on Agricultural Soil Microbiome Members Represented by Metagenomically Assembled Genomes (MAGs) and Their Predicted Plant-Beneficial Genes. Genes.

[21] Griffiths B.S., Philippot L. (2013). Insights into the resistance and resilience of the soil microbial community. FEMS Micro.-Biol..

[22] Degens B.P., Harris J.A. (1997). Development of a physiological approach to measuring the catabolic diversity of soil microbial communities. Soil Biol. Biochem..

[23] Nkongolo K.K., Narendrula-Kotha R. (2020). Advances in monitoring soil microbial community dynamic and function. J. Appl. Genet..

[24] Degens B.P., Schipper L.A., Sparling G.P., Vojvodic-Vukovic M. (2000). Decreases in organic C reserves in soils can reduce the catabolic diversity of soil microbial communities. Soil Biol. Biochem..

[25] Aon M.A., Cabello M.N., Sarena D.E., Colaneri A.C., Franco M.G., Burgos J.L., Cortassa S.I. (2001). Spatio-temporal patterns of soil microbial and enzymatic activities in an agricultural soil. Appl. Soil Ecol..

[26] Lin Q., Brookes P.C. (1999). An evaluation of the substrate-induced respiration method. Soil Biol. Biochem..

[27] Campbell C.D., Chapman S.J., Cameron C.M., Davidson M.S., Potts J.M. (2003). A rapid microtiter plate method to measure carbon dioxide evolved from carbon substrate amendments so as to determine the physiological profiles of soil microbial communities by using whole soil. Appl. Env. Microbiol..

[28] Fierer N., Wood S.A., de Mesquita C.P.B. (2021). How microbes can, and cannot, be used to assess soil health. Soil Biol. Biochem..

[29] Kaiser H.F. (1974). An index of factorial simplicity. Psychometrika.

[30] De Brogniez D., Ballabio C., Stevens A., Jones R.J.A., Montanarella L., van Wesemael B. (2015). A map of the topsoil organic carbon content of Europe generated by a generalized additive model. Eur. J. Soil Sci..

[31] Olayemi O.P., Schneekloth J.P., Wallenstein M.D., Trivedi P., Calderón F.J., Corwin J., Fonte S.J. (2022). Soil macrofauna and microbial communities respond in similar ways to management drivers in an irrigated maize system of Colorado (USA). Appl. Soil Ecol..

[32] Rowell M.J. (1995). Colorimetric method for CO_2_ measurement in soils. Soil Biol. Biochem..

[33] Cookson W.R., Murphy D.V., Roper M.M. (2008). Characterizing the relationships between soil organic matter components and microbial function and composition along a tillage disturbance gradient. Soil Biol. Biochem..

[34] Sandén T., Zavattaro L., Spiegel H., Grignani C., Sandén H., Baumgarten A., Tiirola M., Mikkonen A. (2019). Out of sight: Profiling soil characteristics, nutrients and bacterial communities affected by organic amendments down to one meter in a long-term maize experiment. Appl. Soil Ecol..

[35] Swallow M.J., Quideau S.A. (2015). A method for determining community level physiological profiles of organic soil horizons. Soil Sci. Soc. Am. J..

[36] Babur E., Dindaroğlu T., Riaz M., Uslu O.S. (2022). Seasonal variations in litter layers’ characteristics control microbial respiration and microbial carbon utilization under mature pine, cedar, and beech forest stands in the Eastern Mediterranean Karstic Ecosystems. Microb. Ecol..

[37] Dacal M., Bradford M.A., Plaza C., Maestre F.T., García-Palacios P. (2019). Soil microbial respiration adapts to ambient temperature in global drylands. Nat. Ecol. Evol..

[38] Bradford M.A., McCulley R.L., Crowther T., Oldfield E.E., Wood S.A., Fierer N. (2019). Cross-biome patterns in soil microbial respiration predictable from evolutionary theory on thermal adaptation. Nat. Ecol. Evol..

[39] Ali R.S., Poll C., Kandeler E. (2018). Dynamics of soil respiration and microbial communities: Interactive controls of temperature and substrate quality. Soil Biol. Biochem..

[40] Wang C., Morrissey E.M., Mau R.L., Hayer M., Piñeiro J., Mack M.C., Marks J.C., Bell S.L., Miller S.N., Schwartz E. (2021). The temperature sensitivity of soil: Microbial biodiversity, growth, and carbon mineralization. ISME J..

[41] Creamer R.E., Stone D., Berry P., Kuiper I. (2016). Measuring respiration profiles of soil microbial communities across Europe using MicroResp™ method. Appl. Soil Ecol..

[42] Attademo A.M., Sanchez-Hernandez J.C., Lajmanovich R.C., Repetti M.R., Peltzer P.M. (2021). Enzyme Activities as Indicators of Soil Quality: Response to Intensive Soybean and Rice Crops. Water Air Soil Pollut..

[43] Dotaniya M.L., Aparna K., Dotaniya C.K., Singh M., Regar K.L. (2019). Role of soil enzymes in sustainable crop production. Enzymes in Food Biotechnology.

[44] Jat H.S., Datta A., Choudhary M., Sharma P.C., Dixit B., Jat M.L. (2021). Soil enzymes activity: Effect of climate smart agriculture on rhizosphere and bulk soil under cereal based systems of north-west India. Eur. J. Soil Biol..

[45] Sun Y., Goll D.S., Ciais P., Peng S., Margalef O., Asensio D., Sardans J., Peñuelas J. (2020). Spatial Pattern and Environmental Drivers of Acid Phosphatase Activity in Europe. Front. Big Data.

[46] Bandick A.K., Dick R.P. (1999). Field management effects on soil enzyme activities. Soil Biol. Biochem..

[47] Mangalassery S., Mooney S.J., Sparkes D.L., Fraser W.T., Sjögersten S. (2015). Impacts of zero tillage on soil enzyme activities, microbial characteristics and organic matter functional chemistry in temperate soils. Eur. J. Soil Biol..

[48] Margalef O., Sardans J., Maspons J., Molowny-Horas R., Fernández-Martínez M., Janssens I.A., Richter A., Ciais P., Obersteiner M., Peñuelas J. (2021). The effect of global change on soil phosphatase activity. Glob. Chang. Biol..

[49] Habteselassie M., Woodruff L., Norton J., Ouyang Y., Sintim H. (2022). Changes in microbial communities in soil treated with organic or conventional N sources. J. Environ. Qual..

[50] Zhao J., Ni T., Li Y., Xiong W., Ran W., Shen B., Shen Q., Zhang R. (2014). Responses of Bacterial Communities in Arable Soils in a Rice-Wheat Cropping System to Different Fertilizer Regimes and Sampling Times. PLoS ONE.

[51] Liu Y.R., Delgado-Baquerizo M., Wang J.T., Hu H.W., Yang Z., He J.Z. (2018). New insights into the role of microbial community composition in driving soil respiration rates. Soil Biol. Biochem..

[52] Tkacz A., Bestion E., Bo Z., Hortala M., Poole P.S. (2020). Influence of plant fraction, soil, and plant species on microbiota: A multikingdom comparison. Mbio.

[53] Canfield D.E., Glazer A.N., Falkowski P.G. (2010). The Evolution and Future of Earth’s Nitrogen Cycle. Science.

[54] Xiao D., He X., Wang G., Xu X., Hu Y., Chen X., Zhang W., Su Y., Wang K., Soromotin A.V. (2022). Network analysis reveals bacterial and fungal keystone taxa involved in straw and soil organic matter mineralization. Appl. Soil Ecol..

[55] Henneron L., Kardol P., Wardle D.A., Cros C., Fontaine S. (2020). Rhizosphere control of soil nitrogen cycling: A key component of plant economic strategies. New Phytol..

[56] Yue H., Banerjee S., Liu C., Ren Q., Zhang W., Zhang B., Tian X., Wei G., Shu D. (2022). Fertilizing-induced changes in the nitrifying microbiota associated with soil nitrification and crop yield. Sci. Total Environ..

[57] Lin H.-C., Huber J.A., Gerl G., Hülsbergen K.-J. (2016). Nitrogen balances and nitrogen-use efficiency of different organic and conventional farming systems. Nutr. Cycl. Agroecosystems.

[58] Masson-Delmotte V., Zhai P., Pirani A., Connors S.L., Péan C., Berger S., Caud N., Chen Y., Goldfarb L., Gomis M.I. (2021). IPCC, 2021: Climate Change 2021: The Physical Science Basis. Contribution of Working Group I to the Sixth Assessment Report of the Intergovernmental Panel on Climate Change.

[59] Lazcano C., Zhu-Barker X., Decock C. (2021). Effects of Organic Fertilizers on the Soil Microorganisms Responsible for N2O Emissions: A Review. Microorganisms.

[60] Vives-Peris V., de Ollas C., Gómez-Cadenas A., Pérez-Clemente R.M. (2019). Root exudates: From plant to rhizosphere and beyond. Plant Cell Rep..

[61] Brolsma K.M., Vonk J.A., Mommer L., Van Ruijven J., Hoffland E., De Goede R.G. (2017). Microbial catabolic diversity in and beyond the rhizosphere of plant species and plant genotypes. Pedobiologia.

[62] Hirte J., Leifeld J., Abiven S., Oberholzer H.R., Mayer J. (2018). Below ground carbon inputs to soil via root biomass and rhizodeposition of field-grown maize and wheat at harvest are independent of net primary productivity. Agric. Ecosyst. Environ..

[63] Bergstrom D.W., Monreal C.M., Tomlin A.D., Miller J.J. (2000). Interpretation of soil enzyme activities in a comparison of tillage practices along a topographic and textural gradient. Can. J. Soil Sci..

[64] Choudhary M., Jat H.S., Datta A., Yadav A.K., Sapkota T.B., Mondal S., Meena R.P., Sharma P.C., Jat M.L. (2018). Sustainable ntensification influences soil quality, biota, and productivity in cereal-based agroecosystems. Appl. Soil Ecol..

[65] Kuzyakov Y. (2002). Factors affecting rhizosphere priming effects. J. Plant Nutr. Soil Sci..

[66] Fita A., Garcia-Martinez M.D., Raigon M.D., Lerma M.D., Moreno E., Rodriguez-Burruezo A. (2014). Peppers: Soil dynamics, root architecture and fruit quality. International Congress. STRATEGIES for Organic and Low Input Agricultures and Their Food Systems.

[67] Ribes-Moya A.M., Morales-Manzo I.I., Aguilar C.L., Raigón M.D., Rodríguez-Burruezo A. (2019). Estudio preliminar de la actividad enzimática fosfatasa alcalina y catalasa en cultivos ecológico y convencional de ecotipos de pimiento (*Capsicum* sp.). XXVII Jornadas Técnicas de SEAE. VI Congreso Valenciano de Agricultura Ecológica.

[68] Hu L., Robert C.A.M., Cadot S., Zhang X., Ye M., Li B., Manzo D., Chervet N., Steinger T., van der Heijden M.G.A. (2018). Root exudate metabolites drive plant-soil feedbacks on growth and defense by shaping the rhizosphere microbiota. Nat. Commun..

[69] Berendsen R.L., Pieterse C.M.J., Bakker P.A.H.M. (2012). The rhizosphere microbiome and plant health. Trends Plant Sci..

[70] Moreau D., Bardgett R.D., Finlay R.D., Jones D.L., Philippot L. (2019). A plant perspective on nitrogen cycling in the rhizosphere. Funct. Ecol..

[71] Schmidt J.E., Kent A.D., Brisson V.L., Gaudín A.C.M. (2019). Agricultural management and plant selection interactively affect rhizosphere microbial community structure and nitrogen cycling. Microbiome.

[72] Oburger E., Jones D.L. (2018). Sampling root exudates—Mission impossible?. Rhizosphere.

[73] Wen Z., White P.J., Shen J., Lambers H. (2021). Linking root exudation to belowground economic traits for resource acquisition. New Phytol..

[74] Wen Z., Li H., Shen J., Rengel Z. (2017). Maize responds to low shoot P concentration by altering root morphology rather than increasing root exudation. Plant Soil.

[75] Iannucci A., Canfora L., Nigro F., De Vita P., Beleggia R. (2021). Relationships between root morphology, root exudate compounds and rhizosphere microbial community in durum wheat. Appl. Soil Ecol..

[76] (2022). Microresp. James Hutton Ltd. Scotland, UK, V3.2. www.microresp.com.

[77] Tabatabai M.A., Bremner J.M. (1969). Use of p-nitrophenyl phosphate for assay of soil phosphate activity. Soil Biol. Biochem..

[78] Tabatabai M.A., Page A.L., Miller R.H., Keeney D.R. (1982). Soil enzymes. Methods of Soil Analysis, Part 2, Chemical and Microbiological Properties.

[79] Trevors J.T., Mayfield C.I., Innis W.E. (1982). Measurement of electron transport systema (ETS) activity in soil. Microb. Ecol..

[80] García C., Hernandez T., Costa F., Ceccanti B., Masciandaro G., Gallardo-Lancho J. (1993). The dehydrogenase activity of soil as an ecological marker in process of perturbed system regeneration. Proceedings of the XI International Symposium of Environmental Biochemistry.

[81] Kandeler E., Gerber H. (1988). Short-term assay of soil urease activity using colorimetric determination of ammonium. Biol. Fert. Soils..

[82] Kandeler E., Stemmer M., Klimanek E.M. (1999). Response of soil microbial biomass, urease and xylanase within particle size fraction to long-term soil management. Soil. Biol. Biochem..

[83] Alef K., Nannipieri P. (1995). Methods in Applied Soil Microbiology and Biochemistry.

[84] Capitán L.F., Avidad R., Fernández M.D., Ariza A. (2004). Sensor de Un Solo Uso Para la Detección y Determinación de Nitrito En Aguas. Patent.

[85] Wei T., Simko V. (2021). R Package ‘Corrplot’: Visualization of a Correlation Matrix (Version 0.92). https://github.com/taiyun/corrplot.

[86] Wickham H., Henry L., Pedersen T., Luciani T., Decorde M., Lise V. (2022). Svglite: An ‘SVG’ Graphics Device. R Package Version 2.1.0. https://CRAN.R-project.org/package=svglite.

[87] Revelle W. (2022). Psych: Procedures for Personality and Psychological Research. R Package Version 2.2.9. https://CRAN.R-project.org/package=psych.

[88] Wickham H., Averick M., Bryan J., Chang W., McGowan L.D., François R., Grolemund G., Hayes A., Henry L., Hester J. (2019). Welcome to the tidyverse. J. Open Source Softw..

[89] Jarek S. (2012). Mvnormtest: Normality Test for Multivariate Variables. R Package Version 0.1-9. https://CRAN.R-project.org/package=mvnormtest.

[90] Raiche G., Magis D. (2022). nFactors: Parallel Analysis and Other Non Graphical Solutions to the Cattell Scree Test. R Package Version 2.4.1.1. https://CRAN.R-project.org/package=nFactors.

[91] Navarro-Gonzalez D., Lorenzo-Seva U. (2021). EFA.MRFA: Dimensionality Assessment Using Minimum Rank Factor Analysis. R Package Version 1.1.2. https://CRAN.R-project.org/package=EFA.MRFA.

[92] Wickham H., François R., Henry L., Müller K. (2022). Dplyr: A Grammar of Data Manipulation. R Package Version 1.0.10. https://CRAN.R-project.org/package=dplyr.

